# Relationship between children’s oral health-related behaviors and their caregiver’s sense of coherence

**DOI:** 10.1186/1471-2458-13-239

**Published:** 2013-03-19

**Authors:** Rong Min Qiu, May CM Wong, Edward CM Lo, Huan Cai Lin

**Affiliations:** 1 Department of Preventive Dentistry, Guanghua School of Stomatology, Guangdong Provincial Key Laboratory of Stomatology, Sun Yat-sen University, 56 Ling Yuan Road West, Guangzhou, Guangdong Province, China; 2 Dental Public Health, Faculty of Dentistry, University of Hong Kong, Hong Kong, 510055, China

**Keywords:** Child, Caregiver, Sense of coherence, Oral health-related behavior, Psychological factor

## Abstract

**Background:**

Sense of coherence (SOC) is hypothesized to be an important psychological factor that enables people to cope with stressors and successfully maintain and improve health. Mother’s SOC has been shown to be an important psychological factor associated with oral health and oral health-related behaviors of adolescents and 11- to 12-year-old children. However, little is known about the relationship between the caregiver’s SOC and oral health-related behaviors of the preschool children. The objective of this study was to investigate the relationship between oral health-related behaviors of 5-year-old children in Southern China and SOC of their caregiver.

**Methods:**

A cross-sectional study was conducted in a randomized sample of 1332 children aged 5 years and their caregivers in Guangzhou, Southern China. Data were collected through questionnaires completed by the caregivers. The Chinese short version of Antonovsky’s SOC scale (13 items) was employed to assess the caregiver’s SOC. The outcome variables were the child’s oral health-related behaviors, including frequency of sugary snack intake, toothbrushing frequency, utilization of dental service, and pattern of dental visits. Multiple logistic regression was used to analyze the relationship between the variables.

**Results:**

No association was found between the children’s sugary snack intake and the mother’s or the father’s SOC. After adjustment for other significant factors related to the child’s oral health-related behaviors, 8.9% of the children whose grandparents (as caregivers) had higher SOC scores had a lower frequency of sugary snack intake, compared with the children whose grandparents had lower SOC scores (OR = 0.61, 95% CI = 0.50–0.73, *p* = 0.008). The other measures of oral health-related behaviors of the child were not significantly associated with the caregiver’s SOC.

**Conclusion:**

Sugary snack intake behavior of the 5-year-old children was not associated with the mother’s or the father’s SOC. It was associated with the SOC of their grandparents, who are a small group of the caregivers in China.

## Background

Oral disease is strongly related to lifestyle. Health promoting lifestyles include infrequent sugars consumption, toothbrushing effectively and regularly and visiting a dentist regularly to prevent and detect oral disease [1,2]. The factors influencing those behaviors have not been successfully identified.

Most investigators believe that oral health knowledge and attitudes are the major determinants and promoters of oral health-related behaviors [3,4]. Moreover, psychological factors, such as health locus of control and self-esteem, also increase an individual’s propensity to execute health-promoting behaviors [5,6]. However, all these factors can be encompassed by the concept of sense of coherence (SOC), which is at the center of the salutogenic model [7,8]. SOC consists of three components: comprehensibility, manageability, and meaningfulness [7]; that is to say, an individual’s SOC reflects the degree to which they view life as comprehensive, manageable, and meaningful. According to the salutogenic model, it is important for people to focus on their resources and their capacity to generate health rather than on the causes of their disease. Stronger SOC leads one to perceive the environment as less stressful, disturbing, and chaotic, and it facilitates the selection of efficacious health behaviors [7]. Thus, it is suggested that SOC can promote a person’s awareness of oral health and that people with stronger SOC usually have better oral health-related behaviors [9].

This hypothesis that SOC is a health-promoting resource is supported by a number of studies on SOC and its relationship to oral health-related behaviors. Adults or adolescent with stronger SOC were likely to brush their teeth at least twice a day to maintain oral health [8,10,11]; higher SOC scores were associated with healthier dietary patterns, especially fewer sweet drinks and lower frequency of snacks [12,13]. Higher SOC scores were also associated with regular dental visits [10,14]. However, not all oral health-related behaviors were found to be associated with an individual’s SOC in certain populations [12,14]. Thus far, the relationship between an individual’s SOC and oral health-related behaviors has not been examined in a Chinese population.

Adopting consistent behavioral habits in childhood takes place at home, and caregivers can be the primary model for children’s behavior. It has been confirmed that caregivers influence their children’s oral health and oral health-related behaviors, mainly through their own oral health-related behaviors, knowledge, and attitudes [15,16]. Recently, the mother’s SOC has been shown to be an important psychological factor associated with children’s oral health and oral health-related behaviors [17,18]. A study by Freire et al. found that adolescents whose mothers had higher level of SOC score had lower level of dental caries and gingival bleeding after probing, and were less likely to visit a dentist mainly only when in trouble, than those whose mothers had lower level of SOC [17]. Another study by da Silva et al. that 11- to 12-year-old children whose mothers had higher SOC scores were more likely to utilize dental services and visit a dentist mainly for check-ups [18]. In addition, one study found that 5-year-old children were more likely to have decayed teeth, dental pulp exposure, or filled teeth if their mothers had low SOC [19]. This result indicates that the mother’s SOC is an important psychological determinant of the oral health status of preschool children. However, little is known about the relationship between the caregiver’s SOC and the oral health-related behaviors of preschool children.

Our study explored the relationship between the caregiver’s SOC and oral health-related behaviors of 5-year-old children in China. It was hypothesized that the children whose caregivers had higher SOC would have better oral health-related behaviors, such as lower frequency of sugary snack intake, more frequent toothbrushing, and using dental care services and visiting a dentist regularly for check-ups, than children whose caregivers had lower SOC.

## Methods

### Study population

A cross-sectional study was carried out from August to December 2011 in 24 randomly selected public and private kindergartens in Guangzhou, the capital of Guangdong Province in southern China. Guangzhou has approximately 12 million inhabitants and is divided into 12 administrative districts. The sample was selected from an overall population of about 100,000 5-year-old children in the city, 98% of whom were enrolled in kindergartens.

The required sample size was determined based on the prevalence of dental caries among 5-year-old children in Guangzhou. In the calculation, the estimated prevalence of dental caries was 60%, which was the finding from the latest national oral health survey [20]. The precision of the estimate was set to have a standard error of 1.5%. The minimum sample size to satisfy the requirements was calculated to be 1067 children.

The study protocol was approved by the Ethics Committee of Guanghua School of Stomatology, Sun Yat-sen University. A two-stage sampling technique was employed. The first stage consisted of a simple random selection of six of the 12 administrative districts in Guangzhou. The second stage consisted of a cluster sampling, in which four kindergartens were randomly selected in each of the selected administrative districts. All the 5-year-old children in the selected kindergartens and their main caregivers were invited to take part in this survey. All caregivers were fully informed of the study purpose in writing, and they were given a free choice as to whether or not they participated. Written informed consent for participation was obtained from the caregivers who decided to take part in the study.

To test data collection procedures and ascertain the applicability of the instruments, a pilot study was conducted on 60 children in a kindergarten that was not selected for the survey.

### Instruments and measures

Data were collected by means of a questionnaire administered to the caregivers. The questionnaire consisted of five parts: questions on the child’s oral health-related behaviors (sugary snack intake habits, toothbrushing habits, and use of dental services); questions on the caregiver’s oral health knowledge and attitudes; the SOC scale; questions on the caregiver’s oral health-related behaviors; and questions on the child’s demographic and socioeconomic background. The questionnaire was distributed by the kindergarten to the caregivers, who then completed the questionnaire at home and returned it to the kindergarten.

### Outcome variables

Four oral health-related behaviors of the 5-year-old children were designated as outcome variables: frequency of sugary snack intake (<once/day vs. ≥once/day); toothbrushing frequency (≤once/day vs. ≥twice/day); having used dental services (yes vs. no); and pattern of dental visit for the children who had visited a dentist (for treating dental problems vs. mainly for check-ups).

### Independent variable

The caregiver’s SOC was measured by the short version of the SOC scale (SOC-13) developed by Antonovsky [21]. The scale consists of 13 items, each of which is scored on a Likert scale, ranging from 1 (“very often”) to 7 (“very seldom or never”). The SOC scale measures three dimensions: comprehensibility (five items); manageability (four items); and meaningfulness (four items). After reversing the scores of the five negatively worded items (items 1, 2, 3, 8, and 13), the scores of the 13 items were added to obtain the overall SOC score. Thus, the SOC score could range from 13 to 91, with higher scores indicating stronger SOC. The SOC-13 scale has been translated into many languages, including Chinese, and extensively used in studies in many countries [21,22]. The Chinese version of SOC-13 scale (C-SOC-13) has been shown to have good reliability and validity as well as discriminatory power [22]. In this sample, Cronbach’s alpha coefficient of the C-SOC-13 scale was 0.80.

### Controlling variables

The child’s demographic background (gender, single child, marital status of parents, caregiver) and socioeconomic background (mother’s education and occupation, father’s education and occupation, family income), the caregiver’s oral health-related behaviors (frequency of sugary snack intake, toothbrushing frequency, utilization of dental services, pattern of dental attendance), and oral health knowledge and attitudes were used as controlling variables. Similarly worded questions were used to assess the oral health-related behaviors of the caregiver and those of the child.

The caregiver’s oral health knowledge was measured by four questions about the causes and prevention of tooth decay and periodontal disease, which had been used in a previous study of Guangdong adults [23]. Each correct answer to a question was given a score of 1, and incorrect or “don’t know” answers were scored 0. A maximum of four correct answers was accepted for each question, so the caregiver could score up to 4 points per question. The overall oral health knowledge score was the sum of the scores of the four questions, which could range from 0 to 16, with higher scores indicating better oral health knowledge.

To explore the caregiver’s attitudes toward oral health, eight statements that had been used in a previous study were selected [23]. These related to dental health beliefs and the importance of oral health, retaining natural teeth, and the use of dental services. The response to each statement was “agree,” “disagree,” or “neither.” A dental attitude score was constructed by counting the total number of statements to which the caregiver showed a positive attitude. The final score could range from 0 to 8, with higher scores indicating a more positive attitude toward oral health.

### Statistical analysis

Data analysis was carried out using SPSS for Windows (version 16.0). The total SOC score was analyzed as a continuous variable. Initially, associations between the independent variable and the outcome variables were assessed by two-sample *t* tests. Chi-square tests and *t* tests were carried out to analyze the relationships between the controlling variables and the outcome variables. Controlling variables with a *p* value of 0.20 or lower were included in the multiple logistic regression analysis. Multiple logistic regression analysis was performed to examine the relative significance of the effects of the independent variable and the controlling variables on the outcome variables in different caregiver groups. The level of significance for all statistical tests was set at 0.05.

## Results

Among the 1440 caregivers of the selected children, 46 did not return the questionnaires and 62 returned uncompleted questionnaires. Among the 62 uncompleted questionnaires, most items in the questionnaires were unanswered. Thus, only 1332 caregivers (1141 mothers, 85.7%; 72 fathers, 5.4%; and 119 grandparents, 8.9%) returned completed questionnaires, and their data were entered into the analysis of the relationship between the caregiver’s SOC and the child’s oral health-related behaviors. Among the children whose caregiver had completed the questionnaire, 731 (54.9%) were boys and 601 (45.1%) were girls.

The caregivers’ total SOC scores ranged from 19 to 91, with a mean of 61.1 and a standard deviation of 10.5. The means and standard deviations of the SOC scores of different caregivers were, respectively, 61.0 and 10.3 for the mothers, 63.9 and 10.2 for the fathers, and 60.9 and 12.1 for the grandparents. There was no statistically significant difference in the total SOC scores among the different caregivers (*p* = 0.065).

The results showed that the caregiver’s SOC was significantly associated with the child’s frequency of sugar snack intake but not with toothbrushing frequency, use of dental care, or pattern of dental visit (Table 1). Children whose caregivers scored higher in SOC had a lower frequency of sugar snack intake than children whose caregivers scored lower in SOC (*p* = 0.020).

**Table 1:** Results of univariate analysis between the child’s oral health-related behaviors and the caregiver’s total SOC (n = 1332)

**Outcome variables**	**n**	**%**	**Total SOC**	***P-value***
			**Mean (SD)**	
Frequency of sugary snack intake				
<once/day	671	50.4	61.8 (10.7)	**0.020**
≥once/day	661	49.6	60.4 (10.2)	
Toothbrushing frequency				
≤once/day	896	67.3	61.4 (10.6)	0.900
≥twice/day	436	32.7	60.6 (10.2)	
Utilization of dental care of children				
No	923	69.3	61.1 (10.6)	1
Yes	409	30.7	61.1 (10.1)	
Pattern of dental attendance				
For treatment of dental problem	348	85.1	60.9 (10.0)	0.400
Mainly for check-up	61	14.9	62.1 (11.0)	

Since the caregiver’s SOC was associated with the child’s frequency of sugary snack intake, further analysis was carried out. As shown in Table 2, it was found that the controlling variables of a single child—caregiver, caregiver’s oral health attitudes, and caregiver’s frequency of sugary snack intake—were significantly associated with the child’s frequency of sugary snack intake. After adjusting for the above controlling variables in multiple logistic regressions separately for mothers, fathers, and grandparents, it was found that only the grandparent’s SOC score was significantly associated with the child’s frequency of sugary snack intake. Children whose grandparents had high SOC were less likely to have a sugary snack intake once or daily (Tables 3, 4, 5). The odds ratio was 0.61 (95% CI = 0.50–0.73, *p* = 0.008) for every 10-unit increase in the SOC score. In addition, the frequency of sugar snack intake of the caregivers was significantly related to the child’s frequency of sugar snack intake: children whose caregivers had more frequent sugary snack intake were also more likely to have more frequent sugary snack intake (all odds ratios >1, *p* < 0.05) (Tables 3, 4, 5).

**Table 2:** Results of univariate analysis between the control variables and the child’s frequency of sugary snack intake (n = 1332)

**Variables**	**Frequency of sugary snack intake**		
	**<once/day**	**≥once/day**	***P-value***
	**n (%)**	**n (%)**	
Gender			
Boy	365 (54.4)	366 (55.4)	0.721*
Girl	306 (45.6)	295 (44.6)	
Single child			
Yes	490 (73.0)	446 (67.5)	0.027*
No	181 (27.0)	215 (32.5)	
Caregiver			
Mother	591 (88.1)	550 (83.2)	0.021*
Father	34 (5.0)	38 (5.8)	
Grandfather or grandmother	46 (6.9)	73 (11.0)	
Caregiver’s frequency of sugary snack intake			
<once/day	545 (81.2)	288 (43.6)	<0.001*
≥once/day	126 (18.8)	373 (56.4)	
Marital status of parents			
Cohabiting	659 (98.2)	650 (98.3)	0.862*
Not cohabiting	12 (1.8)	11 (1.7)	
Mother’s education			
≥college graduated	300 (44.7)	305 (46.1)	0.599*
≤high school graduated	371 (55.3)	356 (53.9)	
Mother’s occupation			
Employer/professional	147 (21.9)	143 (21.6)	0.817*
Employee/non-professional	395 (58.9)	399 (60.4)	
Unemployed	129 (19.2)	119 (18.0)	
Father’s education			
≥college graduated	327 (48.7)	328 (49.6)	0.746*
≤high school graduated	344 (51.3)	333 (50.4)	
Father’s occupation			
Employer/professional	223 (33.2)	211 (31.9)	0.465*
Employee/non-professional	426 (63.5)	420 (63.6)	
Unemployed	22 (3.3)	30 (4.5)	
Family monthly income (per-capital)			
≥5000 RMB	253 (37.7)	271 (41.0)	0.457*
2000-4999 RMB	284 (42.3)	262 (39.6)	
<2000 RMB	134 (20.0)	128 (19.4)	
	Mean(SD)	Mean(SD)	
Caregiver’s oral health knowledge score	9.3 (3.4)	9.0 (3.9)	0.161**
Caregiver’s oral health attitude score	6.3 (1.5)	6.1 (1.5)	0.016**

(* by Chi-square test; ** by *t*-test).

**Table 3:** Results of multiple logistic regression analysis between the mother’s SOC score and the child’s frequency of sugary snack intake (n = 1141)

**Variables**	**Frequency of sugary snack intake**	
	(<once/day vs. ≥once/day)*	
	**Adjusted OR**	***P-value***
	**(95% CI)**	
Mother’s SOC score (per 10 units)	-	-
Single child		
Yes	1	
No	1.37 (1.04-1.82)	**0.03**
Mother’s frequency of sugary snack intake		
<once/day	1	
≥ once/day	5.74 (4.39-7.51)	**<0.001**
Mother’s oral health knowledge score	-	-
Mother’s oral health attitude score	-	-

* in the multiple logistic regression analysis, “< once/day” was set as the reference category.

**Table 4:** Results of multiple logistic regression analysis between the father’s total SOC score and the child’s frequency of sugary snack intake (n = 72)

**Variables**	**Frequency of sugary snack intake**	
	(<once/day vs. ≥once/day)*	
	**Adjusted OR**	***P-value***
	**(95% CI)**	
Father’s SOC score (per 10 units)	-	-
Single child		
Yes		
No	-	-
Father’s frequency of sugary snack intake		
<once/day	1	
≥once/day	8.97 (2.97-27.16)	**<0.001**
Father’s oral health knowledge score	-	-
Father’s oral health attitude score	-	-

* in the multiple logistic regression analysis, “<once/day” was set as the reference category.

**Table 5:** Results of multiple logistic regression analysis between the grandparent’s SOC score and the child’s daily frequency of sugary snack intake (n = 119)

**Variables**	**Frequency of sugary snack intake**	
	(<once/day vs. ≥ once/day)*	
	**Adjusted OR**	***P-value***
	**(95% CI)**	
Grandparent’s SOC score (per 10 units)	0.61 (0.50-0.73)	**0.01**
Single child		
Yes		
No	-	-
Grandparent’s frequency of sugary snack intake		
<once/day	1	
≥once/day	2.53 (1.08-5.95)	**0.033**
Grandparent’s oral health knowledge score	-	-
Grandparent’s oral health attitude score	-	-

* in the multiple logistic regression analysis, “<once/day” was set as the reference category.

From the above results, it appeared that there could be an interaction effect between the different groups of caregivers and their SOC scores. Thus, a separate multiple logistic regression was performed by considering such an interaction effect. However, the interaction effect was found to be insignificant (*p* = 0.137).

## Discussion

This cross-sectional study showed that the frequency of sugary snack intake by 5-year-old children was not associated with the mother’s or father’s SOC. It was associated with the SOC of their grandparents (as caregivers), with a lower frequency of intake among children whose grandparent had stronger SOC. The other measures of oral health-related behaviors of the child were not significantly associated with the caregiver’s SOC.

The caregiver’s SOC was significantly connected with the child’s sugary snack intake frequency in univariate analyses, but such an association was not found after adjustment for other associated factors in the mother group and father group. This suggests that different groups of caregivers and their SOC might influence the children’s sugary snack intake habits interactively; we had assessed for the interaction effect, but no significant interaction was found. The probable reason may be the imbalance of the sample size in the different groups of caregivers (85.7% for mothers, 5.4% for fathers, and 8.9% for grandparents). This result is consistent with that of Freire et al., whereby the mother’s SOC was not associated with an adolescent’s daily frequency of sugar intake [17]. In our study, we found that the children’s sugary snack intake frequency was significantly related to the mother’s or father’s sugary snack intake frequency. This indicates that the children’s sugary snack intake frequency may be influenced by the parents’ sugary snack intake behavior rather than their SOC. It has been demonstrated in other studies that the frequent consumption of sweets by parents is associated with such behavior in their children [15,16].

Our results show that the children whose grandparents (as caregivers) scored higher in SOC had lower frequency of sugar snack intake. It was reported that individuals with higher SOC scores had higher intake of fruits but lower intakes of energy, total and saturated fat, sucrose, and sweets, which indicates that individuals with a higher SOC score were better in “healthy” food choices [13]. Excessive intake of sugar products not only increases the risk of early childhood caries [24], it also increases the risk of childhood obesity [25]. Thus, the present study could provide new, useful information regarding the promotion of oral health and general health among Chinese children who are looked after by grandparents. However, it should be noted that the number of grandparents whose SOC was related to the child’s sugary snack intake habits represents a small sample; the association between the grandparents’ SOC and children’s sugary snack intake habits needs to be confirmed in a further study with a large sample.

An association between the child’s toothbrushing habit or use of dental care service and the caregiver’s SOC was not observed in any of the grandparent, mother or father groups. The results indicate that not all of the oral health-related behaviors of the children were related to the caregiver’s SOC, which is consistent with the findings of a previous study of Freire et al. [17], in which mother’s SOC was shown to be related to the oral health-related behaviors of 15-year-old adolescents mainly in the pattern of dental attendance; however, they found no relation to other oral health-related behaviors, such as daily frequency of sugar intake, daily between-meal frequency of sugar intake, and daily toothbrushing frequency. Similar results were also found in some studies on individuals’ SOC [12,14]. The reason for the SOC not being related to all oral health-related behaviors has not been clarified. In this study, we found that regardless of who the caregiver was, their intake frequency of sugary snacks was significantly related to that of the children. Therefore, we inferred that caregivers’ SOC might not fully explain the children’s oral health-related behaviors and that such behaviors are still mainly influenced by the caregivers’ oral health behaviors.

Previous reports have shown an association between the mother’s SOC and her child’s utilization of dental services and dental visit pattern [17,18]. Such an association was not found in the present study. This discrepancy may be related to the differences in the age of the study subjects and dental care systems. The children in the present study were 5 years old, whereas previous studies were conducted on older children or adolescents. Another possible explanation relates to differences in the dental care systems in different countries. Although dental services have improved substantially in China over the last several decades, the availability of dental services to young children and their accessibility are still limited. In China, there is a severe shortage of pediatric dentists, which limits young children’s use of dental services. In our study, 30.7% of the children had utilized dental care services, and only 14.9% of them visited the dentist regularly for check-ups. This result suggests that most caregivers neglect the importance of the dentist in children’s oral health. With China’s rapid economic development and further development in health and oral health care delivery systems, children’s use of dental services may change in coming years.

Since mothers are the major group of caregivers in China, more attention should be given to the mother’s impact on the child’s oral health behavior. However, in the present study, none of the children’s oral health-related behaviors was found to be associated with the SOC of the mother. Thus, the practical significance of the caregiver’s SOC in children’s oral health-related behavior does not appear to be so evident in China.

Notwithstanding the above remarks, some further points should be noted with respect to this study. Few studies of SOC and dental health have been conducted in Chinese populations since the C-SOC-13 scale was created [26,27], and no study has addressed SOC and oral health-related behaviors. Thus, the manner in which SOC influences Chinese health behaviors demands further research. However, we should observe that the frequency of children using dental services (30.7%), visiting dentists regularly for check-ups (14.9%), and brushing their teeth twice a day or more (32.7%) was relatively low. In a longitudinal study, increasing adolescents’ SOC by interventions was shown to lead to better future toothbrushing behavior [28]; such a longitudinal study should be conducted to examine this phenomenon in young children.

The findings of this study should be considered in relation to its methodological strengths and limitations. First, a strong point of this study was the use of a large random sample, which included various socioeconomic groups. Second, it is one of the first studies to evaluate the caregiver’s SOC in relation to oral health-related behaviors of young children. This study was limited in that it relied on the caregivers’ reporting of the children’s oral health-related behaviors, which may have resulted in reporting bias [28]. However, caregiver reports of children’s oral health-related behaviors have generally been found to be reliable [29]. Additionally, the data in this study were cross-sectional, and this precludes making inferences about causal relationships between the caregiver’s SOC and the child’s oral health behaviors. Future work on this topic should adopt a longitudinal approach.

## Conclusion

Sugary snack intake behavior of the 5-year-old children was not associated with the mother’s or the father’s SOC. It was associated with the SOC of their grandparents, who are a small group of the caregivers in China.

## Competing interests

The authors declare that they have no competing interests.

## Authors’ contributions

RMQ: design of the study, data collection and writing of the manuscript. MCMW: critical review of the data analysis and results. ECML: design of the study and revision of the manuscript. HCL: design of the study, training and supervision of fieldworkers, critical revision of the manuscript for important intellectual content. All the authors read and approved the final manuscript.

## Pre-publication history

The pre-publication history for this paper can be accessed here:

http://www.biomedcentral.com/1471-2458/13/239/prepub
